# *Clostridium cellulovorans* Proteomic Responses to Butanol Stress

**DOI:** 10.3389/fmicb.2021.674639

**Published:** 2021-07-21

**Authors:** Paolo Costa, Giulia Usai, Angela Re, Marcello Manfredi, Giuseppe Mannino, Cinzia Margherita Bertea, Enrica Pessione, Roberto Mazzoli

**Affiliations:** ^1^Structural and Functional Biochemistry, Laboratory of Proteomics and Metabolic Engineering of Prokaryotes, Department of Life Sciences and Systems Biology, University of Turin, Turin, Italy; ^2^Centre for Sustainable Future Technologies, Fondazione Istituto Italiano di Tecnologia, Turin, Italy; ^3^Department of Applied Science and Technology, Politecnico di Torino, Turin, Italy; ^4^Center for Translational Research on Autoimmune and Allergic Diseases, Università del Piemonte Orientale, Novara, Italy; ^5^Department of Translational Medicine, Università del Piemonte Orientale, Novara, Italy; ^6^Plant Physiology Unit, Department of Life Sciences and Systems Biology, University of Turin, Turin, Italy

**Keywords:** heat shock proteins, Hfq chaperone, stringent response, ATPase, butyrate

## Abstract

Combination of butanol-hyperproducing and hypertolerant phenotypes is essential for developing microbial strains suitable for industrial production of bio-butanol, one of the most promising liquid biofuels. *Clostridium cellulovorans* is among the microbial strains with the highest potential for direct production of *n*-butanol from lignocellulosic wastes, a process that would significantly reduce the cost of bio-butanol. However, butanol exhibits higher toxicity compared to ethanol and *C. cellulovorans* tolerance to this solvent is low. In the present investigation, comparative gel-free proteomics was used to study the response of *C. cellulovorans* to butanol challenge and understand the tolerance mechanisms activated in this condition. Sequential Window Acquisition of all Theoretical fragment ion spectra Mass Spectrometry (SWATH-MS) analysis allowed identification and quantification of differentially expressed soluble proteins. The study data are available via ProteomeXchange with the identifier PXD024183. The most important response concerned modulation of protein biosynthesis, folding and degradation. Coherent with previous studies on other bacteria, several heat shock proteins (HSPs), involved in protein quality control, were up-regulated such as the chaperones GroES (Cpn10), Hsp90, and DnaJ. Globally, our data indicate that protein biosynthesis is reduced, likely not to overload HSPs. Several additional metabolic adaptations were triggered by butanol exposure such as the up-regulation of V- and F-type ATPases (involved in ATP synthesis/generation of proton motive force), enzymes involved in amino acid (e.g., arginine, lysine, methionine, and branched chain amino acids) biosynthesis and proteins involved in cell envelope re-arrangement (e.g., the products of Clocel_4136, Clocel_4137, Clocel_4144, Clocel_4162 and Clocel_4352, involved in the biosynthesis of saturated fatty acids) and a redistribution of carbon flux through fermentative pathways (acetate and formate yields were increased and decreased, respectively). Based on these experimental findings, several potential gene targets for metabolic engineering strategies aimed at improving butanol tolerance in *C. cellulovorans* are suggested. This includes overexpression of HSPs (e.g., GroES, Hsp90, DnaJ, ClpC), RNA chaperone Hfq, V- and F-type ATPases and a number of genes whose function in *C. cellulovorans* is currently unknown.

## Introduction

The consequences of massive exploitation of fossil fuels on global warming and climate changes have prompted research toward alternative energy sources with lower environmental impact. Biofuels produced by microbial fermentation of plant biomass have attracted substantial interest based on their potential to benefit current environmental, economic, and societal issues (Lynd, 2017). It has been estimated that plant biomass provides 10% of global primary energy and, within this, cellulosic feedstocks are the most abundant and least expensive (Lynd, 2017).

*Clostridium cellulovorans*, an anaerobic, mesophilic, cellulolytic bacterium (Sleat et al., 1984) is among the most promising candidates for industrial production of cellulosic biofuels, with particular reference to *n*-butanol (hereinafter referred to simply as butanol). Butanol (four carbon chain) has a longer carbon backbone than other established biofuels such as ethanol (two carbons) or methanol (one carbon), which gives it fuel properties more similar to that of gasoline, such as high combustion energy, low volatility and corrosivity (Liu et al., 2013a). Pure butanol can be fed to spark ignited engines without any modification, whereas ethanol must be blended with gasoline (Campos-Fernández et al., 2012). *C. cellulovorans* potential to ferment all the main plant polysaccharides, namely cellulose, hemicelluloses and pectins (Aburaya et al., 2015, 2019) represents an advantage over other well established plant-degrading microorganisms such as *Clostridium thermocellum*, *Clostridium cellulolyticum*, or *Thermoanaerobacterium saccharolyticum* which show more restricted substrate panel (Saxena et al., 1995; Demain et al., 2005). The most abundant *C. cellulovorans* fermentation products are organic acids (e.g., butyrate, formate, acetate), ethanol, H_2_ and CO_2_ (Sleat et al., 1984). Although wild type *C. cellulovorans* cannot biosynthesize butanol, its production in this microorganism has recently been enabled by introducing a single heterologous alcohol/aldehyde dehydrogenase (Yang et al., 2015). In fact, most metabolic reactions leading to conversion of acetyl-CoA to butyrate are in common with butanol biosynthesis. Butyrate production is widespread in solventogenic clostridia such as *C. acetobutylicum* and *C. beijerinckii* but is absent in most cellulolytic clostridia such as *C. thermocellum* and *C. cellulolyticum* (Mazzoli and Olson, 2020). By using metabolic engineering, recombinant *C. cellulovorans* strains have been obtained which can produce about 4 g/L and 4.96 g/L of butanol through direct fermentation of crystalline cellulose (Bao et al., 2019) and alkali-extracted corn cobs (Wen et al., 2020), respectively. These butanol titers are the highest reported so far for direct fermentation of plant biomass using a single microorganism (Mazzoli and Olson, 2020).

However, butanol displays higher cell toxicity than other biofuels (e.g., ethanol) (Ingram, 1976) which limits fermentation titers. In addition, butanol separation generally requires two distillation columns instead of one (Vane, 2008), which increases capital costs. Native butanol producers (i.e., *C. acetobutylicum* and *C. beijerincki*) typically can attain 15–20 g/L butanol titer (Tomas et al., 2003; Nicolaou et al., 2010; Chen and Liao, 2016). However, *C. cellulovorans* cannot grow in media containing more than 8 g/L butanol (Yang et al., 2015). Recently, only moderate improvement of *C. cellulovorans* tolerance to butanol has been obtained by adaptive evolution (Wen et al., 2019). Although continuous solvent extraction from fermentation medium or two-phase (organic-aqueous) fermentation systems can be used to circumvent solvent toxicity, their cost threatens process viability (Heipieper et al., 2007; Huang et al., 2010; Dürre, 2011). The development of strains with superior solvent tolerance is highly desirable for sustainable production of biofuels (Nicolaou et al., 2010). Enhancement of microbial solvent tolerance can be achieved through different strategies belonging to two main paradigms: (i) “random” approaches, such as random mutagenesis (Liu et al., 2012), whole genome shuffling (Mao et al., 2010) and adaptive laboratory evolution (Liu et al., 2013b) and; (ii) “rational” approaches based on targeted gene modification (e.g., overexpression of genes involved in solvent tolerance) (Tomas et al., 2003; Borden and Papoutsakis, 2007; Xu et al., 2020).

Different experimental approaches have been employed to identify genes involved in solvent resistance such as the construction of genomic or deletion libraries or the use of transcriptomic and proteomic analyses (Borden and Papoutsakis, 2007; Alsaker et al., 2010; Mazzoli, 2012; Venkataramanan et al., 2015). The relatively high butanol cell-toxicity is mainly attributed to its partition coefficient (logP = 1), namely its higher ability to intercalate within the lipid bilayer of biological membranes and increase their fluidity with respect to less hydrophobic biofuels (e.g., ethanol) (Heipieper et al., 2007). From this standpoint, solvent effect on cells is similar to that of temperature upshift (heat shock). Solvents mostly affect the structure and functions of biological membranes, thus compromising vital processes such as energy generation and nutrient transport (Bowles and Ellefson, 1985; Heipieper et al., 2007). Butanol was shown to inhibit membrane-bound ATPases, partially or completely abolish the membrane ΔpH (Bowles and Ellefson, 1985; Gottwald and Gottschalk, 1985; Terracciano and Kashket, 1986) and Δψ (Terracciano and Kashket, 1986), lower intracellular pH and ATP concentration (Bowles and Ellefson, 1985; Huang et al., 1986; Terracciano and Kashket, 1986), besides interfering with active uptake of glucose and other nutrients (Bowles and Ellefson, 1985). With regards to native butanol producers, most research aimed at understanding mechanisms of butanol tolerance refers to *C. acetobutylicum* (Tomas et al., 2004; Borden and Papoutsakis, 2007; Alsaker et al., 2010; Wang et al., 2013b). It has been reported that butanol-challenged *C. acetobutylicum* increases the ratio of saturated/unsaturated fatty acids in the cell membrane, likely to balance the fluidity increase caused by solvent (a response also referred as homeoviscous adaptation) (Borden and Papoutsakis, 2007). Furthermore, responses to butanol challenge have been investigated in a number of other microorganisms which have been proposed for recombinant butanol production owing to their native higher tolerance to butanol, e.g., lactic acid bacteria (Winkler and Kao, 2011; Liu et al., 2021; Petrov et al., 2021) and *Pseudomonas putida* (del Cuenca et al., 2016), or genetic tractability, e.g., *Escherichia coli* (Rutherford et al., 2010) and *Synechocystis* sp. (Tian et al., 2013) with respect to Clostridia. Apart from adaptation mechanisms directly targeted to restore membrane function (which may also include adjustment of the protein content; Weber and De Bont, 1996), responses to solvents generally include up-regulation of heat shock proteins (HSPs) and may comprise overexpression of solvent efflux pumps and changes in cell size and shape (Heipieper et al., 2007; Nicolaou et al., 2010).

However, microbial responses to solvent stress are complex and species-related. The present investigation aimed at digging into *C. cellulovorans* responses to butanol challenge and identifying genes possibly involved in tolerance to this chemical. A comparative proteomic analysis was performed on *C. cellulovorans* cultures grown in butanol-supplemented medium and in control medium (i.e., without butanol) leading to identification of 307 differentially expressed proteins. This protein dataset will help future rational metabolic engineering strategies for obtaining butanol-hypertolerant strains to be exploited in large scale production of this biofuel.

## Materials and Methods

### Growth Conditions

*Clostridium cellulovorans* was grown anaerobically as described previously (Usai et al., 2020) with D-glucose as the main carbon source (5 g/L). Additionally, butanol was supplemented at a concentration ranging from 1 g/L to 8 g/L, when required. Inocula were grown until exponential growth phase and then transferred into 500 mL butyl-stoppered bottles containing 400 mL of medium supplemented with the selected butanol concentration. Cultures were incubated at 37°C in continuous agitation (200 rpm). For each growth condition, three independent cultures were performed. Hourly, microbial growth was assessed through estimation of the optical density at 600 nm (OD_600 nm_).

### Analytical Techniques

For determination of total protein cell content, 1 mL culture samples were centrifuged at 16,000 *g* for 10 min. Cell pellets were re-suspended in 100 μL of 0.2 M NaOH and incubated at 100°C for 10 min. Protein quantification was performed by using the Bradford reagent (Sigma-Aldrich Inc., St. Louis, MO, United States) following the manufacturer’s instructions. Bovine serum albumin was used as the standard. In addition, in order to calculate the biomass amount of each culture, a conversion factor, previously determined in our laboratory, was used: the ratio between proteins (g) and biomass (g) is 0.261.

Glucose content in cell free supernatants was measured through glucose oxidase/peroxidase reaction as previously described (Usai et al., 2020).

Acetate, lactate, formate, butyrate, ethanol, and butanol in culture supernatants were quantified by high-pressure liquid chromatography (HPLC, Agilent Technologies 1200 series), equipped with an Aminex HPX-87H column (Bio-Rad, Hercules, CA, United States). The detection was performed with a UV–Vis detector set at 210 nm for organic acids and a refractive index detector for alcohols. The mobile phase was 5 mM H_2_SO_4_ at a flux of 0.5 mL/min and a temperature of 50°C.

Total intracellular ATP was quantified by using CellTiter-Glo^®^ One Solution (Promega Corporation, Madison, WI, United States) as previously described (Usai et al., 2020).

### Proteomic Analysis

#### Cytosolic Proteins Extraction and in Solution Protein Digestion

Biomass samples (four biological replicates for each growth condition) were collected during the exponential growth phase (4 h after culture inoculation) (4,000 × *g*, 4°C, 20 min). Cell pellets were washed twice with 0.9% NaCl. The soluble proteins were extracted as previously described (Usai et al., 2020). Briefly, pellets were resuspended in 6 ml of 2% (v/v) SDT-lysis buffer [2 mM Tris-HCl, 0.4% (w/v) SDS, pH 7.6], with 100 mM DTT, incubated 10 min at 95°C and centrifuged (4,000 × *g*, 20 min). The supernatant was centrifuged again (10 min, 10,000 × *g*). Proteins were precipitated from supernatants by methanol chloroform method, then resuspended in 25 mM NH_4_HCO_3_ with 0.1% SDS (w/v). Protein concentration was measured by the 2-D Quant kit (GE Healthcare, Chicago, IL, United States), following the manufacturer’s instructions. Protein samples were subjected to in-solution digestion with trypsin (Sequence Grade, Promega Corporation, Madison, WI, United States) as previously described (Usai et al., 2020). Trypsin activity was stopped by adding pure formic acid and digests were dried by Speed Vacuum (Dalla Pozza et al., 2018). Samples were desalted on the Discovery^®^ DSC-18 solid phase extraction (SPE, Sigma-Aldrich Inc., St. Louis, MO, United States) and then analyzed as previously described (Martinotti et al., 2016).

#### SWATH-MS Analysis

The digested protein samples were analyzed by SWATH-MS (Sequential Window Acquisition of all Theoretical fragment ion spectra Mass Spectrometry) (Collins et al., 2017). The LC–MS/MS analyses were performed as previously described (Usai et al., 2020). The LC system was interfaced with a 5600+ TripleTOF system (SCIEX, Concord, Canada). Samples used to generate the SWATH-MS spectral library were subjected to data-dependent acquisition (DDA) and cyclic data independent analysis (DIA) of the mass spectra, applying a 25-Da window as previously reported (Carbonare et al., 2018). Three technical replicates for each sample were employed for DIA analysis and MS data were acquired with Analyst TF 1.7 (SCIEX, Concord, Canada).

#### Protein Database Search and Protein Quantification

The MS files were searched using Protein Pilot v. 4.2 (SCIEX, Concord, Canada) and Mascot v. 2.4 (Matrix Science Inc., Boston, MA, United States), as previously described (Usai et al., 2020). The UniProt/Swiss-Prot reviewed database containing *C. cellulovorans* proteins (NCBI_Clostridium_cellulovorans743B, version 30,102,017, 4278 sequence entries) was adopted. The label-free quantification was performed as previously described (Usai et al., 2020). Briefly, the integration of the extracted ion chromatogram of all the unique ions for a given peptide was performed by PeakView 2.0 and MarkerView 1.2. (Sciex, Concord, ON, Canada). An integrated assay library was built with SwathXtend, using DDA acquisitions data (protein FDR threshold of 1%). Six peptides per protein and six transitions per peptide were extracted from the SWATH files and peptides with FDR lower than 1% were used for the label-free quantification. Shared peptides were excluded as well as peptides with modifications.

#### Statistical Analysis and Differentially Expressed Proteins

Differentially expressed proteins were identified by performing statistical analysis on the relative abundances of proteins quantified in the different conditions. Quantified proteins were subjected to *t*-test using MarkerView 1.2 software (Sciex, Concord, ON, Canada) and proteins were considered differentially expressed for *p*-value < 0.05 and fold change > 1.5 or <0.67 (Dematteis et al., 2020).

### Protein Classification

The genes encoding the differentially expressed proteins were classified according to the functional annotations of the precomputed Clusters of Orthologous Groups of proteins (COGs) stored in the EggNOG (evolutionary genealogy of genes: Non-supervised Orthologous Groups) (Huerta-Cepas et al., 2019) database^1^. EggNOG is a public resource of orthology relationships, gene evolutionary histories and functional annotations. In particular, the genes encoding differentially regulated proteins were aligned to eggNOG 5.0 database using eggnog-mapper (v2.0.1) (Huerta-Cepas et al., 2017). The functional information from the COGs obtained for each query gene were transferred to the query gene.

The percentage of up-/and down-regulated proteins was computed for each COG-extracted functional category. Fold enrichment of each category in up- and down-regulated proteins was computed as the ratio between the number of up- or down-regulated proteins belonging to a certain category and the number of proteins in the proteome belonging to the category.

Differentially expressed proteins assigned to each COG category were further investigated by querying the Uniprot database (Bateman et al., 2021) for functional annotations or searching the literature. Information was deemed relevant for interpreting our results if it concerned *C. cellulovorans* genes encoding differentially regulated proteins or genes which were found to be their best hits by performing standard protein BLAST analysis^2^. All main speculations were reliant on direct or indirect evidences retrieved in literature.

### Quantitative Real-Time PCR Analysis (qRT-PCR)

#### Total RNA Isolation

For gene expression analysis, cell pellets of *C. cellulovorans* (three biological replicates for each growth condition) were harvested (4,000 × *g*, 4°C, 20 min) 4 h after culture inoculation (i.e., at the same point of the exponential growth phase analyzed by comparative proteomics), washed twice with 0.9% NaCl, and extracted combining RNAprotect^®^ Bacterial Reagent (Qiagen, Hilden, Germany) and RNeasy Mini Kit^®^ (Qiagen, Hilden, Germany). Briefly, an equal amount of cell pellet (5 × 10^8^ cells) was transferred in a sterile plastic tube in which 1 mL of RNAprotect^®^ Bacterial Reagent was added. The mixture was incubated for 5 min at room temperature (RT) then centrifuged for 10 min at 5000 *g*. Following centrifugation, the supernatant was removed, and 200 μL of lysis buffer (10 mM Tris-HCl, 1 mM EDTA adjusted to pH 8 and supplemented with 1 mg/mL lysozyme and 2 μL β-mercaptoethanol) was added. The samples were mixed by vortexing, incubated for 5 min at RT and vortexed every 10 s for 2 min. Then, total RNA was isolated using the RNeasy Mini Kit^®^, following manufacturer’s instructions. Total RNA quantity was measured using an UV/visible spectrophotometer Ultrospec 3000 (Amersham Biosciences, Sweden) while the quality was checked by 1% (w/v) agarose gel electrophoresis.

#### cDNA Synthesis and Quantitative Real-Time (qPCR) Analysis

One μg of total RNA was reverse-transcribed using Maxima H Minus First Strand cDNA Synthesis Kit (Thermo Fisher Scientific, United States), following the manufacturer’s instructions. The resulting cDNA was employed as a template for quantitative real-time PCR using the QuantStudio 3 Real-Time PCR system (Thermo Fisher Scientific, United States). For each qPCR reaction primers 0.3 μM, 4.1 μL of nuclease-free H_2_O and 5 μL of SYBR-Green I (Maxima SYBR Green/ROX qPCR Master Mix 2X, Thermo Fisher Scientific, United States) were used and the thermal conditions were as reported in Campobenedetto et al. (2021). The gene encoding RpsJ/30S ribosomal protein S10 (Clocel_3734) was selected as reference (housekeeping) gene by using the Normfinder software (De Spiegelaere et al., 2015) and used to calibrate and normalize qPCR results. Relative expression levels of genes were calculated by using the Pfaffl method (Pfaffl, 2001).

Primers for both target and reference genes, listed in Supplementary Table 1, were designed using Primer3 software (Untergasser et al., 2012).

## Results and Discussion

### *C. cellulovorans* Growth Parameters at Different Butanol Concentrations

**FIGURE 1:**
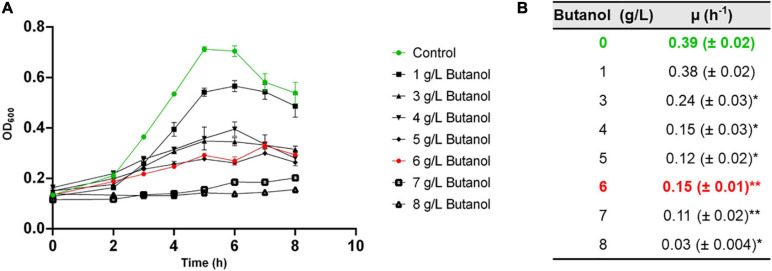
Growth kinetics **(A)** and specific growth rate (μ, **B**) of *C. cellulovorans* grown in media supplemented with different butanol concentration. Bars represent standard errors. Data are the averages of three biological replicates. Asterisks indicate values that significantly (* *p* < 0.05; ** *p* < 0.005) differ from that measured in the control condition (no added butanol).

The effect of supplementing different butanol concentrations (1–8 g/L) on *C. cellulovorans* growth and metabolism was determined. Growth was only slightly affected by 1 g/L butanol (Figure 1A), while higher butanol concentration led to progressive reduction of growth efficiency. At 6 g/L butanol, the maximum biomass reached was about 50% of that obtained in control conditions (no added butanol). Consistently, butanol supplementation negatively affected the specific growth rate (μ) (Figure 1B). These results are consistent with those obtained by Yang et al. (2015).

Eventually, these analyses were aimed at finding the most suitable butanol concentration for studying butanol-stress response in *C. cellulovorans*. For this purpose, a butanol concentration that significantly inhibits growth efficiency but allows enough biomass production for proteomic analyses is ideal. Cultures with 4–7 g/L butanol showed comparable growth rate and maximum biomass and were considered suitable for proteomic analyses. Among these, cultures with 6 g/L butanol were finally chosen.

### Fermentative Metabolism of *C. cellulovorans* in Medium Supplemented With 6 g/L Butanol

**FIGURE 2:**
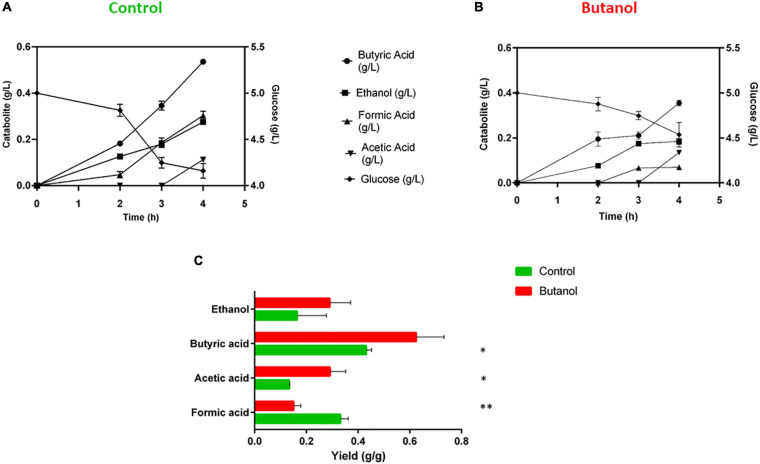
Glucose consumption and catabolite production of *C. cellulovorans* grown in control condition (no added butanol, **A**) or in butanol-supplemented medium **(B)**. **(C)** Fermentation end-product yield (g per g of consumed glucose). Data are the mean of triplicate measurements. Bars represent standard errors. Asterisks indicate values that significantly (^∗^*p*-value < 0.05; ^∗∗^*p*-value < 0.01) differ between control (green) and butanol-supplemented (red) culture conditions.

Almost a double amount (0.84 ± 0.18 g/L) of glucose was consumed by *C. cellulovorans* grown in control condition as compared to 6 g/L butanol-enriched cultures (0.46 ± 0.03 g/L) after 4 h (Figure 2). This seems consistent with differences in the specific growth rate measured in the two conditions (Figure 1B) and with previous studies reporting butanol inhibition of glucose uptake in other clostridia (Bowles and Ellefson, 1985; Tomas et al., 2004; Alsaker et al., 2010; Venkataramanan et al., 2015; Sedlar et al., 2019). However, it is worth noting that butanol inhibition on *C. cellulovorans* growth is more important than that on glucose consumption (Figures 1, 2). Butanol-supplemented cultures showed specific glucose consumption about 2-fold higher than control cultures. As regards catabolite production, formate titer significantly decreased (from 0.30 g/L to 0.07 g/L) in butanol-supplemented condition (Figures 2A,B) as well as formate yield (from 0.33 g/g glucose consumed to 0.15 g/g glucose consumed) (Figure 2C). The opposite pattern was observed for acetate and butyrate yield: 0.29 g/g acetate and 0.62 g/g butyrate were measured in butanol-supplemented condition, while only 0.13 g/g acetate and 0.43 g/g butyrate were produced in the control condition (Figure 2C). No significant difference in ethanol yield was measured between the two growth conditions. No significant butanol consumption (<0.35 g/L) was observed in butanol-supplemented cultures (data not shown). In *C. cellulovorans*, pyruvate may have three metabolic fates: (i) oxidation by pyruvate ferredoxin oxidoreductase (PFOR), leading to production of acetyl-CoA and reduced ferredoxin (possibly used for hydrogen production); (ii) conversion to formate and acetyl-CoA by pyruvate formate lyase (PFL) and; (iii) reduction to lactate by lactate dehydrogenase (LDH) with concomitant consumption of NAD(P)H. According to fermentation end-product determination, LDH reaction accounts for very minor carbon flux, since no detectable lactate amounts were produced, and most pyruvate flux is expectedly taken in charge by PFL and PFOR reactions. The decrease of formate yield in butanol-challenged cultures is indicative of an increase in the carbon flux through the PFOR reaction in this condition. Higher amount of reducing equivalents generated by the PFOR reaction may also have promoted acetyl-CoA-to-butyrate pathway [which requires 3 NAD(P)H per butyrate molecule]. In a number of studies on cellulolytic clostridia, it has been reported that acetate is preferentially accumulated in conditions promoting slower growth (Riederer et al., 2011; Munir et al., 2016; Badalato et al., 2017). This observation finds confirmation in the present study, in which acetate yield was higher in butanol-supplemented cultures.

### Proteome Analysis: Identification of Differentially Expressed Proteins in 6 g/L Butanol-Challenged Cultures

**FIGURE 3:**
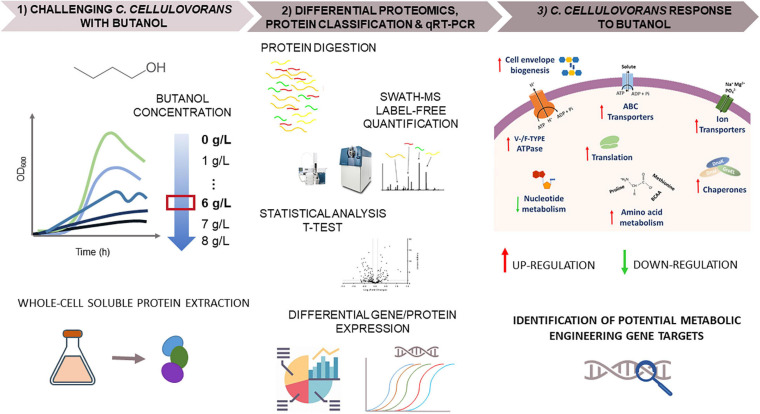
Schematic overview of the proteomic workflow used to study *C. cellulovorans* responses to butanol stress. (1) *C. cellulovorans* was grown in media supplemented with different concentrations of butanol (0–8 g/L). Whole-cell soluble protein extraction was performed on *C. cellulovorans* cells grown in control (0 g/L butanol) or butanol-challenged (6 g/L butanol) cultures. (2) Differential proteomics were analyzed through Sequential Window Acquisition of all Theoretical fragment ion spectra Mass Spectrometry (SWATH-MS). Phylogenetic protein classification by COGs (Clusters of Orthologous Groups of proteins) was performed and qRT-PCR was used to confirm differential gene expression of a gene pool. (3) Butanol-challenged *C. cellulovorans* metabolism was described by discussing the differentially expressed proteins (up- and down-regulated in butanol-stressed bacteria). This information led to identification of target genes for rational metabolic engineering strategies to obtain a butanol-hypertolerant *C. cellulovorans* strain for large scale bio-production.

**FIGURE 4:**
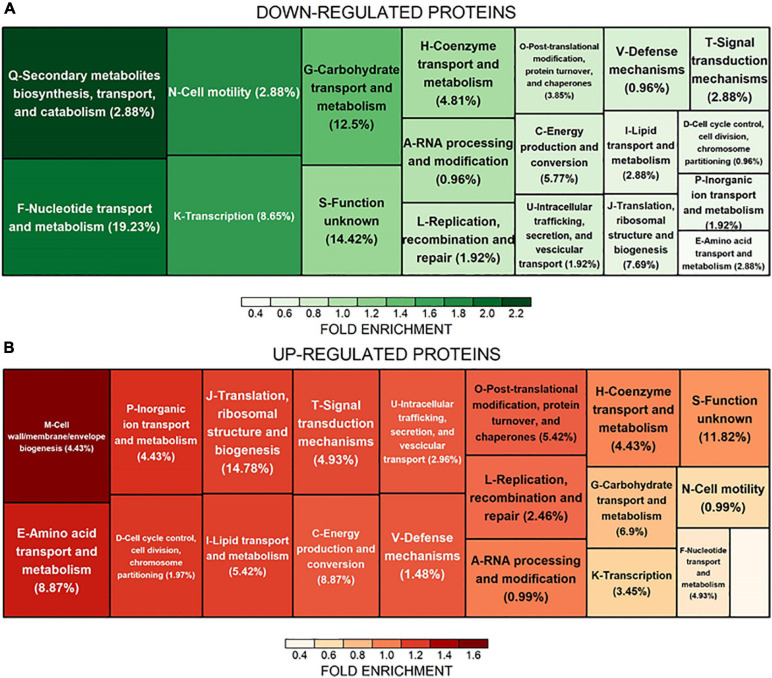
COG categories overrepresentation in differentially expressed proteins. The treemap displays the fold enrichment of each COG category in **(A)** down-regulated and **(B)** up-regulated proteins. The size and color of the rectangles are proportional to the registered fold enrichment. The proportion of regulated proteins that are annotated to each COG category is shown in brackets.

Biomass samples (four biological replicates for each growth condition) for proteomic analyses were harvested 4 h after culture inoculation, that is during exponential growth phase (Figure 1A). A schematic overview of the proteomic workflow used to study *C. cellulovorans* responses to butanol stress is depicted in Figure 3. Proteins showing a fold change (FC) ≥ 1.5 or FC ≤ 0.67 (*p-value* ≤ 0.05) in butanol-treated cultures versus control ones were considered as differentially expressed. The analysis identified 203 up-regulated and 104 down-regulated proteins (Table 1 and Supplementary Table 2) in butanol-stressed cells. The complete list of identified proteins with protein coverage is reported in Supplementary Tables 3–5. The study data are available via ProteomeXchange with the identifier PXD024183. Differentially expressed proteins were classified according to the Clusters of Orthologous Groups (COGs) (Galperin et al., 2015). It is worth noting that a significant amount of *C. cellulovorans* genes encode proteins with still unknown function (Tamaru et al., 2010), namely 12% of the up-regulated and 14% of the down-regulated proteins (that is 24 and 15 proteins, respectively) identified in the present investigation (Figure 4). Apart from these proteins, the overexpressed proteins were predominantly mapped to COG categories representing translation (COG category J, 30 proteins), amino acid metabolism (COG category E, 18 proteins), energy production and conservation (COG category C, 18 proteins) and molecular chaperones (COG category O, 11 proteins) which globally account for almost 50% of the up-regulated proteins. More than 50% of the identified down-regulated proteins belongs to nucleotide metabolism (COG category F, 20 proteins), carbohydrate metabolism (COG category G, 13 proteins), transcription (COG category K, 9 proteins) and translation (COG category J, 8 proteins) related groups. Similar findings have previously been reported in butanol-challenged *C. acetobutylicum*, at least as regards proteins belonging to COG categories C, E, F, and G (Venkataramanan et al., 2015).

**TABLE 1:** List of the most highly differentially expressed proteins quantified in the present study for each COG category.

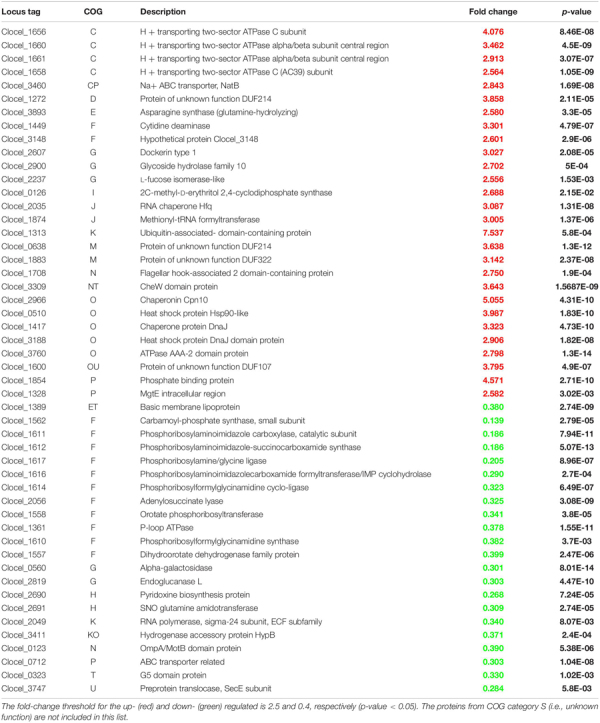

Expression profile of thirteen key genes was validated by means of qRT-PCR analysis (Table 2). Selected genes encode proteins that are representative of eight different COG categories (C, E, F, I, J, KT, O, P). Mostly genes encoding proteins highly up-regulated in butanol-supplemented cultures were considered, but also those coding for slightly (Clocel_1554) and strongly (Clocel_1562) down-regulated proteins were included in the selection. Biomass samples for this analysis were collected at the same time point for which proteomic analysis was carried out. For most of the genes selected (nine), qRT-PCR confirmed the expression profile determined by proteomic analyses (Table 2). However, four genes showed an opposite expression trend, i.e., Heat shock protein Hsp90-like (Clocel_0510), Asparagine synthase (glutamine-hydrolyzing) (Clocel_3893), ATP:guanido phosphotransferase (Clocel_3761) and acyl-ACP thioesterase (Clocel_4352). In general terms, this result is not aberrant, since similar ratio (about 30%) of inconsistencies between proteomic and transcriptomic data has been observed by previous investigations on other microorganisms grown under butanol stress (Tian et al., 2013; Venkataramanan et al., 2015). In the study by Venkataramanan et al. (2015), comparative analysis of proteomic against two sets of transcriptomic data obtained with two different approaches (microarray and RNA-seq) was performed in *C. acetobutylicum* and opposite patterns between proteomic and transcriptomic results were ascribed to post-transcriptional regulation of gene expression. The present results indicate an overall good quality of our proteomic data but suggest that post-transcriptional regulation mechanisms may also occur in *C. cellulovorans*. Additional comments to specific genes/proteins will be given in the following sections.

**TABLE 2:** Validation of protein expression profiles obtained by proteomic analysis through quantitative real-time PCR analysis (qRT-PCR).

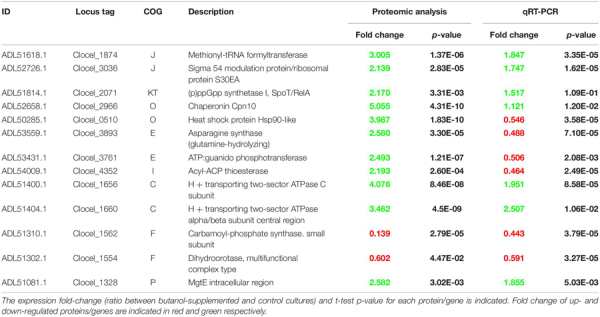

Differentially expressed proteins found in the present investigation will be thoroughly discussed in the next sections.

#### Proteins Involved in Protein Translation, Folding and Degradation

Cell exposure to solvents, such as butanol, induces effects which are similar to heat shock, including protein unfolding and aggregation, that is sometimes reported as proteotoxic stress (Heipieper et al., 2007; Schäfer et al., 2020). Cell responses to solvent shock generally include modulation of the expression of proteins with housekeeping functions such as protein translation (e.g., to replace denatured proteins), folding, and degradation (Patakova et al., 2018). Proteins belonging to COG category J (translation, including ribosome structure and biogenesis) and O (molecular chaperones and related functions) have generally been found as among the most differentially expressed under butanol stress (Alsaker et al., 2010; Fu et al., 2013; Tian et al., 2013; Venkataramanan et al., 2015; del Cuenca et al., 2016; Sedlar et al., 2019). In addition, genome sequencing of a butanol-tolerant mutant of *C. acetobutylicum* detected a high number of point mutations in genes encoding rRNAs which likely affect the structure and function of ribosomes (Bao et al., 2014). Studies on *E. coli* have indicated that short-chain alcohols inhibit transcription and translation processivity by proning ribosomes to misreading errors and stalling and causing aberrant termination of transcription (Haft et al., 2014).

##### Proteins involved in translation and ribosome structure

**FIGURE 5:**
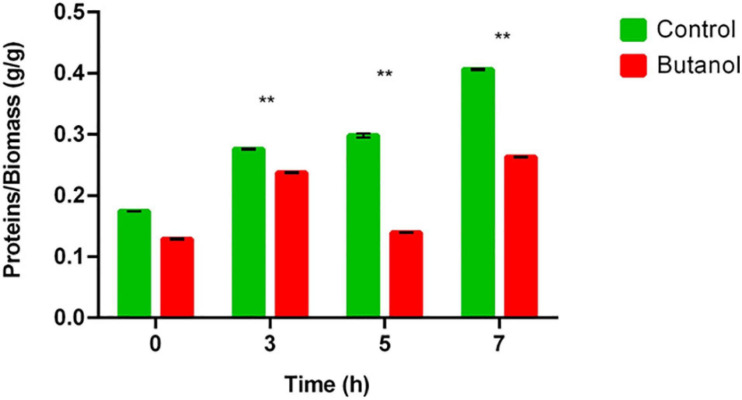
Protein content of cells grown in control (green) or butanol-supplemented medium (red) in different growth phases. Data are the mean of triplicate measurements. Asterisks indicate values that significantly (**p-value < 0.01) differ between the two growth conditions.

In the present study, COG category J is one of the most represented (15%) among the proteins overexpressed by butanol-challenged *C. cellulovorans*. These proteins include 9 aminoacyl-tRNA synthetases, 14 ribosomal proteins, a translation initiation factor and a methionyl-tRNA formyltransferase which are directly involved in protein biosynthesis (Table 1 and Supplementary Table 2). These results appear inconsistent with similar studies performed on a number of other bacterial models such as *C. acetobutylicum* (Alsaker et al., 2010; Venkataramanan et al., 2015), *C. beijerinckii* (Sedlar et al., 2019), *Staphylococcus warneri* (Fu et al., 2013), and *Synechocystis* sp. (Tian et al., 2013) which reported an enrichment of proteins belonging to COG category J among those down-regulated by butanol challenge. As far as we know, the present study is the first reporting extensive up-regulation of proteins belonging to COG category J in microorganisms challenged with butanol. Interestingly, qRT-PCR results, although obtained on a restrained number of genes belonging to COG category J, further support proteomic evidence (Table 2). Down-regulation of genes/proteins involved in translation and ribosome structure has been correlated with growth inhibition observed under butanol stress (Alsaker et al., 2010; Tian et al., 2013; Venkataramanan et al., 2015; Sedlar et al., 2019). Although *C. cellulovorans* growth was inhibited by butanol supplementation, category J proteins were mostly up-regulated in this microorganism, thus indicating that there is no obvious correlation between the two phenomena. However, protein content of butanol-challenged *C. cellulovorans* was significantly lower than that measured in control conditions all throughout the growth kinetics, thus indicating that protein translation is less efficient under solvent stress (Figure 5). The latter result seems consistent with up-regulation of (p)ppGpp synthetase I (SpoT/RelA, Clocel_2071) in butanol-supplemented *C. cellulovorans* cultures, which suggests an increase of cellular levels of (p)ppGpp that mediates the so called stringent response (Schäfer et al., 2020). (p)ppGpp is involved in bacterial response to multiple stresses, including nutrient limitation, heat shock and ethanol (Harty et al., 2019; Schäfer et al., 2020). Increased levels of (p)ppGpp have been reported to modulate many aspects of bacterial physiology and metabolism, including inhibition of transcription, translation, GTP biosynthesis, DNA replication and microbial growth (Potrykus and Cashel, 2008; Schäfer et al., 2020). Clocel_2071 encodes a bifunctional protein consisting of both (p)ppGpp synthetase and hydrolase domains, however, it is likely that Clocel_2071 up-regulation under butanol stress actually leads to intracellular accumulation of (p)ppGpp. Recently, a link between butanol tolerance and stringent response has also been reported in *Lactobacillus mucosae* (Liu et al., 2021). A very recent study reported that (p)ppGpp mainly inhibits translation in *Bacillus subtilis*, for instance by binding to the translation initiation factor IF-2 and other ribosome-associated GTPases (Schäfer et al., 2020). In addition, *C. cellulovorans* up-regulated proteins under butanol shock include ribosome hibernation promoting factor (Hpf, Clocel_3036) which mediates the formation of functionally inactive 100S ribosomes in bacteria (namely ribosome dimers formed through interactions between their 30S subunits), thus contributing to decrease translation rate (Yoshida and Wada, 2014). Overexpression of Hpf has been considered as a hallmark of the stringent response activation in *B. subtilis* [its transcription is activated by (p)ppGpp] (Schäfer et al., 2020). Altogether, these data suggest that translation is diminished in butanol-challenged *C. cellulovorans*, consistent with other investigations indicating that under proteotoxic stress microorganisms decrease translation as a mean to reduce the load on the cellular protein quality control systems (HSPs) (Schäfer et al., 2020). The reason why several ribosomal proteins and other proteins involved in translation were up-regulated in butanol-challenged *C. cellulovorans* currently remains elusive.

The most up-regulated protein in COG class J is the RNA chaperone Hfq (Clocel_2035, FC = 3.09). Hfq is a global modulator of the activity of small regulatory RNAs (sRNAs) (Cho et al., 2014). sRNAs act as major posttranscriptional regulators of gene expression through binding with target mRNAs and Hfq promotes this binding thus acting as an enhancer (Faigenbaum-Romm et al., 2020). In bacteria, sRNAs are involved in regulation of gene expression regulation in response to a variety of stresses, including solvent stress (Venkataramanan et al., 2013; Pei et al., 2017). Growth of *hfq*-deleted *Acinetobacter baumannii* was dramatically compromised when exposed to stresses including ethanol and temperature (Kuo et al., 2017). In addition, the latter study demonstrated that Hfq is involved in regulation of a number of stress-related genes including *groEL*. Ethanol has been shown to induce up-regulation of *hfq* gene in *Zymomonas mobilis* (Cho et al., 2017). Mutations in *hfq* gene have been identified in isobutanol hypertolerant *E. coli* strains obtained by adaptive evolution suggesting the role of this gene in adaptation to solvents (Minty et al., 2011). Butanol stress has been reported to induce Hfq up-regulation and differential expression of 84 sRNAs (several of which were predicted to be Hfq targets) in *C. acetobutylicum* (Venkataramanan et al., 2013).

##### Molecular chaperones

**FIGURE 6:**
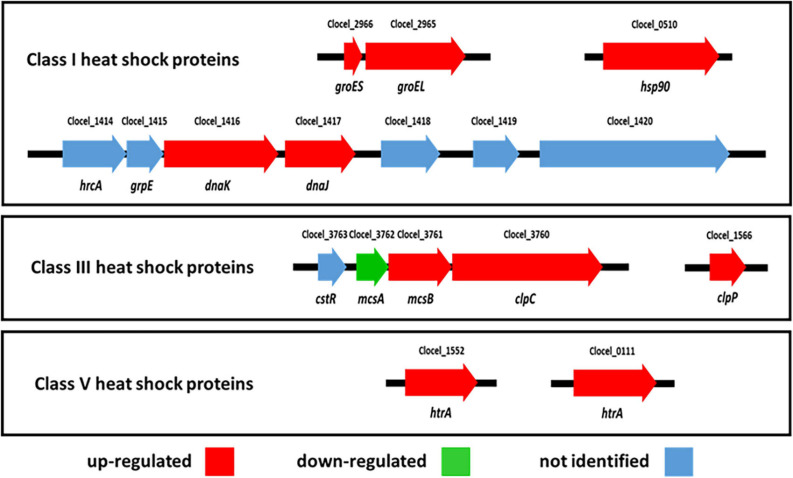
The expression of several heat shock proteins (HSPs) is affected in butanol-challenged *C. cellulovorans*. The gene loci encoding the main *C. cellulovorans* HSPs are indicated in red or green depending on if their protein products were up-regulated or down-regulated in butanol challenged cultures. Products of the genes indicated in blue were not identified in the present study.

Proteins overexpressed in butanol-supplemented *C. cellulovorans* cultures encompass 11 members of the COG category O (molecular chaperones and related functions). HSPs comprise two major classes, namely chaperones binding to denatured or misfolded proteins and promoting refolding to their native structure, and ATP-dependent proteases which catalyze hydrolysis of irreversibly damaged proteins (Schumann, 2016). In Gram positive bacteria, HSPs have mainly been studied in *B. subtilis*, and divided in six classes based on mechanisms that regulate their expression (Schumann, 2003). Some of the proteins identified here, namely the molecular chaperones GroEL (Cpn60, Clocel_2965), GroES (Cpn10, Clocel_2966), DnaJ (Clocel_1417) and DnaK (Clocel_1416), belong to class I (i.e., HrcA-regulated) HSPs which traditionally include the bicistronic *groE* and the heptacistronic *dnaK* operons (Figure 6; Schumann, 2003). The structure of these operons in *C. cellulovorans* is highly conserved with respect to *B. subtilis*. Up-regulation of *groE* operon and of *dnaKJ* genes upon butanol exposure has also been observed in *C. acetobutylicum* and *C. beijerinckii* (Tomas et al., 2004; Sedlar et al., 2019). Consistent with the latter studies, up-regulated HSPs in butanol-challenged *C. cellulovorans* also include the molecular chaperone Hsp90 (HtpG, Clocel_0510) (Figure 6). Transcription of Class I HSP genes is regulated by CIRCE (controlling inverted repeat of chaperone expression) sequences which are found in the DNA region upstream of the operon (Schumann, 2003). A search for the CIRCE motif (TTAGCACTC-N9-GAGTGCTAA) in the *C. cellulovorans* genome identified three exact matches, namely upstream of the *groE* and *dnaK* operons and *hsp90* gene. This confirms previous findings on *C. acetobutylicum* (Tomas et al., 2003) and indicates that these genes/gene clusters belong to the same regulon even in *C. cellulovorans* (although *hsp90* had previously been located in a different HSP class, at least in *B. subtilis*) (Schumann, 2003). Curiously, qRT-PCR analysis confirmed up-regulation of GroES also at the transcript level, but indicates a down-regulation of Hsp90 mRNA (Table 2). *C. cellulovorans* overexpressed proteins under butanol challenge also include class III (i.e., CtsR-regulated) HSPs, namely ClpP (Clocel_1566) and ClpC (ATPase AAA-2 domain protein, Clocel_3760). ClpC and ClpP form the ClpCP ATP-dependent protease (Figure 6) (Schumann, 2003). In addition, two proteins (Clocel_3761, Clocel_3762) which do not belong to COG category O but are encoded by the same *clpC* operon, hence belong to the CstR regulon, are differentially expressed in the conditions tested (Figure 6). In particular, the product of Clocel_3761 is up-regulated by butanol stress, while the protein encoded by Clocel_3762 is down-regulated in the same condition. The proteins encoded by Clocel_3761 and Clocel_3762 show high sequence identity with McsB (44%) and McsA (37%) from *B. subtilis*, respectively. McsAB are modulators of the CtsR activity, however, McsA is thought to stabilize CtsR binding to DNA (thus repressing regulated genes), while McsB phosphorylates CstR making it inactive and promotes its proteolysis by ClpCP (thus enabling transcription of genes under CstR regulation) (Schumann, 2003, 2016). Opposite expression patterns of these proteins observed in butanol-challenged *C. cellulovorans* seem therefore consistent with activation of transcription of genes in the CstR regulon. Both peptidase S1 and S6 chymotrypsin/Hap (Clocel_0111, FC 2.22) and HtrA2 peptidase (Clocel_1552, FC 2.18) from *C. cellulovorans* show some sequence identity with *C. acetobutylicum* HtrA (40 and 37%, respectively). HtrA belongs to class V (i.e., CssRS-regulated) HSPs and is thought to be a membrane-anchored protease acting on non-native proteins within or on the outer face of the cytoplasmic membrane (Schumann, 2003). These results essentially confirm those previously obtained on butanol-challenged *C. acetobutylicum* (Alsaker et al., 2010). Furthermore, up-regulation of HSPs has been frequently observed in butanol-challenged microorganisms (Rutherford et al., 2010; Fu et al., 2013; Liu et al., 2021).

#### Amino Acid Metabolism and Transport

Almost all (namely 18 out of 21) the differentially expressed proteins involved in amino acid metabolism and transport identified in this study were up-regulated in butanol-stressed *C. cellulovorans*. COG category E was the second most represented class among overexpressed proteins in butanol-challenged *C. cellulovorans*. A similar observation was reported on butanol-exposed *S. warneri* (Fu et al., 2013). Up-regulated enzymes are involved in the biosynthesis of a number of amino acids including proline and arginine (Clocel_2734, Clocel_3150, Clocel_1668), lysine (Clocel_1978, Clocel_3115), methionine (Clocel_1764, Clocel_2896, Clocel_3040), and branched chain amino acids (BCAA) (Clocel_1324, Clocel_1325, Clocel_0493) (Table 1 and Supplementary Table 2). In addition, up-regulated proteins include four aminotransferases (Clocel_1948, Clocel_2059, Clocel_2390, Clocel_3812).

The role of amino acids in the cellular stress response is a well-known concern. Increase in pyrroline-5-carboxylate reductase and intracellular levels of proline in response to butanol stress has been observed in *B. subtilis* (Mahipant et al., 2017). A mutant proline-accumulating *Saccharomyces cerevisiae* is more tolerant to ethanol stress (Takagi et al., 2005). Experimental evidence suggesting a role of L-proline as inhibitor of protein aggregation and chaperone for protein folding has been reported (Samuel et al., 2008). Additional proline functions include: (i) osmoprotection (Zaprasis et al., 2015); (ii) improvement of stability or solubility of hydrophobic macromolecules and soluble proteins (Schobert and Tschesche, 1978; Samuel et al., 1997) and; (iii) reduction of solvent-induced membrane disorder (Takagi et al., 2005). In yeasts, arginine acts both as cryo- (Morita et al., 2002) and osmo-protectant (Noti et al., 2018). A possible role of amino acids as osmoprotectants in butanol stress has been hypothesized (Wang et al., 2013a). A study on *E. coli* indicated that limitations in cellular levels of methionine and methionyl-tRNA could be among the mechanisms of ethanol toxicity and that methionine supplementation could increase ethanol tolerance in this bacterium (Haft et al., 2014). The authors speculated that the main effect of methionine limitation is a reduction of the protein translation efficiency, owing to longer ribosome stalling at non-start AUG codons. Up-regulation of proteins involved in methionine biosynthesis in butanol-challenged *C. cellulovorans* suggests that higher cellular levels of methionine could be present in these conditions. It is therefore tempting to hypothesize that a phenomenon similar to that observed in *E. coli* could also occur in *C. cellulovorans*. In addition, methionine is involved in multiple other functions, e.g., it contributes to oxidative stress response and participates in several methyltransferase reactions (Rodionov et al., 2004; Luo and Levine, 2009). Up-regulation of genes involved in BCAA biosynthesis by bacteria exposed to butanol has already been reported in *C. acetobutylicum* (Alsaker et al., 2010; Janssen et al., 2012) and increased intracellular levels of BCAA have actually been measured in this bacterium under butanol stress (Wang et al., 2016). The relationship between the increased levels of BCAA and butanol stress has generally been referred to the role of BCAA as primers for the synthesis of branched-chain fatty acids and the role of the latter in modulating cell membrane fluidity (Mansilla et al., 2004; Alsaker et al., 2010; Wang et al., 2016). In *B. subtilis*, incorporation of branched-chain fatty acids in cell membrane has been identified as an alternative strategy to change membrane fluidity with respect to saturating/desaturating fatty acids (Mansilla et al., 2004). It is therefore possible to hypothesize that increased levels of enzymes involved in the biosynthesis of BCAA in *C. cellulovorans* are involved in strategies to cope with altered membrane fluidity caused by butanol.

Differential expression of proteins involved in amino acid biosynthesis and in particular their up-regulation and/or increased intracellular concentration of amino acids has been reported in a number of microorganisms (e.g., *C. acetobutylicum*, *B. subtilis, E. coli*) exposed to butanol and other alcohol stress (Wang et al., 2013a, 2016; Venkataramanan et al., 2015; Mahipant et al., 2017; Li et al., 2019). The present study indicates that *C. cellulovorans* response to butanol involves at least some elements of the stringent response, which typically includes up-regulation of amino acid biosynthesis mediated by the CodY transcriptional regulator. To understand if CodY could also be involved in up-regulation of amino acid biosynthetic enzymes in butanol-challenged *C. cellulovorans*, the 15-nucleotide CodY canonical consensus motif AATTTTCWGAAAATT (Belitsky and Sonenshein, 2013) was searched throughout the *C. cellulovorans* genome. Through this analysis, 1, 9, 126, or 1386 putative CodY binding sites were identified depending on if 0, 1, 2, or 3 mismatches were allowed. It is worth remembering that CodY binding sequences, their location (upstream or within a gene coding sequence), and CodY regulation mechanisms (either negative or positive) may be highly variable. This makes computational approaches for predicting putative CodY-regulated genes hardly conclusive (Belitsky and Sonenshein, 2013) as it was the case for the present study. However, it might be worth testing this hypothesis in future investigations.

#### Cell Envelope Structure and Biogenesis

The primary cell target of solvent toxicity is the cell envelope (Heipieper et al., 2007). As other solvents and hydrophobic compounds, butanol compromises cell envelope structure and function, including cell wall and membrane thus requiring activation of repair responses (Mazzoli et al., 2011). Among proteins overexpressed in butanol-challenged *C. cellulovorans*, eight enzymes were involved in different stages of peptidoglycan biosynthesis or remodeling, that is glucose-1-phosphate thymidylyltransferase (Clocel_3025), UDP-*N*-acetylglucosamine pyrophosphorylase (Clocel_3808), two D-alanine/D-alanine ligases (Clocel_3085 and Clocel_0693), mur ligase (domain of unknown function DUF1727, Clocel_2906), peptidoglycan transferase (Clocel_2098), cell wall hydrolase/autolysin (Clocel_2663), and a zinc metalloprotease (Clocel_1781) (Sangshetti et al., 2017). Previous evidence that UDP-*N*-acetylglucosamine pyrophosphorylase is overexpressed under butanol stress has been reported in *S. warneri* and *Synechocystis* sp. PCC 6803 (Fu et al., 2013; Zhu et al., 2013). Enzymes involved in cell wall recycling and/or autolysis in clostridia have been associated with butanol tolerance (Webster et al., 1981; Van Der Westhuizen et al., 1982; Croux et al., 1992). D-alanine/D-alanine ligase was found as part of the ethanol tolerance response in *Oenococcus oeni* (Silveira et al., 2004). Studies on *Clostridium beijerinckii* NRRL B-59 have suggested that increased tolerance to butanol might be associated to peptidoglycan thinning (Linhová et al., 2010).

Because of its chaotropic effects, butanol increases biological membrane fluidity (Heipieper et al., 2007). It has been previously reported that Clostridia increase the saturated fatty acid content of cell membrane (thus decreasing its fluidity) when they are exposed to butanol (Baer et al., 1987, 1989; Huffer et al., 2011; Isar and Rangaswamy, 2012). In the present study, five proteins involved in the biosynthesis of saturated fatty acids (Clocel_4136, Clocel_4137, Clocel_4144, Clocel_4162, and Clocel_4352) and two enzymes likely involved in phospholipid biosynthesis (Clocel_1331, Clocel_1338) were identified among the proteins up-regulated by butanol. Curiously, one of the two acyl carrier proteins (Clocel_4143) encoded by the *C. cellulovorans* genome was down-regulated. Globally, these data indicate that fatty acid biosynthesis is improved in butanol-challenged cells, which is likely related to a change in fatty acid composition of the cell membrane. Extensive up-regulation of enzymes involved in fatty acid biosynthesis has been reported in a number of bacteria upon butanol stress, such as *S. warneri* (Fu et al., 2013). Up-regulated proteins in butanol challenged *C. cellulovorans* also include two enzymes involved in the synthesis of terpene precursor isopentenyl-PP (Clocel_0126, Clocel_1782).

In addition, butanol-challenged *C. cellulovorans* overexpresses a couple of proteins belonging to the MreB/Mrl family (Clocel_3042 and Clocel_2768) which are involved in cell shape regulation (Egan et al., 2020). Changes in cell shape and/or size have been observed in several bacteria under butanol stress (Heipieper et al., 2007; Fletcher et al., 2016). Cell elongation and filamentous growth was reported for *E. coli* (Fletcher et al., 2016), while an increase of the cell size of *P. putida* and *Enterobacter* sp. (Neumann et al., 2005) and a decrease of the cell size of *Pseudomonas taiwanensis* (Halan et al., 2017) were observed. These modifications likely affect the surface-to-volume ratio of cells. For instance, a reduced surface-to-volume ratio is thought to diminish butanol entry into the cell (Heipieper et al., 2007).

#### Membrane Transport

Differentially expressed proteins identified in this study include 16 components of membrane transporters. Most of them (14, namely Clocel_0638, Clocel_0903, Clocel_1272, Clocel_1328, Clocel_1355, Clocel_1356, Clocel_1854, Clocel_2887, Clocel_2598, Clocel_3460, Clocel_3854, Clocel_3857, Clocel_4100, Clocel_4152) were up-regulated in butanol-challenged *C. cellulovorans*. Currently, the protein product of Clocel_0638 does not have any associated function, however, it shows a very high sequence identity (73–75%) with some ATP-dependent permeases found in other *Clostridia* (e.g., Uniprot entry U2DAG1, T0N9N5 and A0A1M6LTB1^3^). Increased levels of membrane transporters have previously been observed in other bacteria under butanol stress or as involved in butanol tolerance (Gao et al., 2017; Yang et al., 2020). As mentioned above, butanol negatively alters cell membrane structure and function, including intrinsic proteins involved in nutrient and ion transport (Bowles and Ellefson, 1985). The chaotropic effect of alcohols leads to increased membrane permeability for ions and other small solutes (Hutkins and Kashket, 1986), which partially or completely abolishes transmembrane ΔpH and Δψ (Bowles and Ellefson, 1985; Gottwald and Gottschalk, 1985; Terracciano and Kashket, 1986; Wang et al., 2005). It therefore might be hypothesized that increased levels of transporters could improve ion pumping outside the cell membrane thus compensating solvent effects and restoring homeostasis. Transporters up-regulated in butanol-stressed *C. cellulovorans* include two cation transporters, namely NatB (Clocel_3460) and MgtE (Clocel_1328). NatB mediates Na^+^ extrusion from cells and was overexpressed by ethanol in *B. subtilis* (Cheng et al., 1997) and possibly involved in ethanol tolerance in *Clostridium phytofermentans* (Tolonen et al., 2015). MgtE is considered among the primary Mg^2+^ transporters in bacteria and involved in Mg^2+^ uptake (Groisman et al., 2013). A mutation in a Mg^2+^ transporter has been described among the genetic traits of an ethanol-hypertolerant *C. phytofermentans* (Tolonen et al., 2015), while Mg^2+^ supplementation has been shown to reduce dissipation of membrane ΔpH caused by butanol in *C. beijerinckii* (Wang et al., 2005). It has been hypothesized that both Na^+^ and Mg^2+^ are employed by bacteria to maintain membrane ΔpH by means of Na^+^/H^+^ antiporters and Mg^2+^-dependent H^+^-translocating ATPases (Wang et al., 2005). A recent study reported that the overexpression of an ABC transporter (ButTM) belonging to the multidrug resistance (MDR) systems led to substantial increase of *C. acetobutylicum* tolerance to butanol (Yang et al., 2020). This raised the hypothesis that ButTM acts as a solvent (butanol, ethanol) extruding pump, similar to solvent efflux pumps found in other bacteria (Patakova et al., 2018), although this role was not confirmed by experimental evidence. A *btrR-btrT-btrM-btrK*-like gene cluster was found in the *C. cellulovorans* genome (Clocel_4202-5) consistently with other clostridia (Yang et al., 2020) although none of these genes was up-regulated in butanol-supplemented cultures. However, another ABC-related transporter up-regulated in the present study (Clocel_2887) shows significant identity with some MDR transporters (COG category V, i.e., defense mechanisms).

Finally, proteins up-regulated by butanol exposure include two components of an ABC transporter putatively involved in dipeptide/oligopeptide transport (Clocel_1355-6). This finding seems consistent with increased requirement of amino acids as suggested by the activation of pathways for amino acid biosynthesis described above.

#### Energy Production and Conversion

Five (out of nine) subunits of a V-type ATPase (V_1_ subunits K, Clocel_1656; C, Clocel_1658; F, Clocel_1659; A, Clocel_1660; and B, Clocel_1661) and three (out of eight) subunits of a F-type ATPase (F_1_ subunits γ, Clocel_3050; α, Clocel_3051; and δ, Clocel_3052) were overexpressed by *C. cellulovorans* cells grown in butanol-supplemented medium. Expression profile of Clocel_1656 and Clocel_1660 was confirmed by qRT-PCR also (Table 2). Interestingly, one of the subunits of the F-type ATPase was down-regulated (i.e., F_0_ subunit B, Clocel_3053). Both enzymes are reversible ATPases/synthetases since they function as molecular motors that hydrolyze or synthesize ATP depending on the physiological conditions (Nakanishi et al., 2019). ATP hydrolysis is used to generate an ion (H^+^ or Na^+^) gradient across the cell membrane, while consumption of electrochemical gradient is used to synthesize ATP (Murata et al., 2005; Nakanishi et al., 2018). Both V- and F-type ATPases share the same overall architecture consisting of a hydrophilic portion (F_1_/V_1_) catalyzing ATP synthesis/hydrolysis and a membrane moiety responsible for ion translocation across the membrane (F_o_/V_o_) (Murata et al., 2005; Nakanishi et al., 2018).

As mentioned above, butanol is known to inhibit membrane-bound ATPases, diminish the membrane ΔpH (Bowles and Ellefson, 1985; Gottwald and Gottschalk, 1985; Wang et al., 2005) and Δψ (Terracciano and Kashket, 1986), and lower intracellular pH (Bowles and Ellefson, 1985; Huang et al., 1986; Terracciano and Kashket, 1986) and ATP concentration (Bowles and Ellefson, 1985). Cytoplasm acidification leads to several cell damages such as enzyme denaturation, alteration of nutrient uptake, oxidative stress, depurination and depyrimidination of DNA, and disruption of amino acid pools (Charoenbhakdi et al., 2016; Ju et al., 2016). Up-regulation of V- and F-type ATPases is likely a strategy to cope with disruption of membrane ΔpH and/or reduction of intracellular ATP pool and restore cell homeostasis. Interestingly, up- (Ghiaci et al., 2013; Tian et al., 2013) or down- (Mao et al., 2011; Fu et al., 2013; Liu et al., 2021) regulation of ATPase expression has been observed upon butanol stress depending on the microbial model. This may be related to the reversible activity of ATPases/synthetases and different physiology of microorganisms studied.

#### Nucleotide Metabolism

**FIGURE 7:**
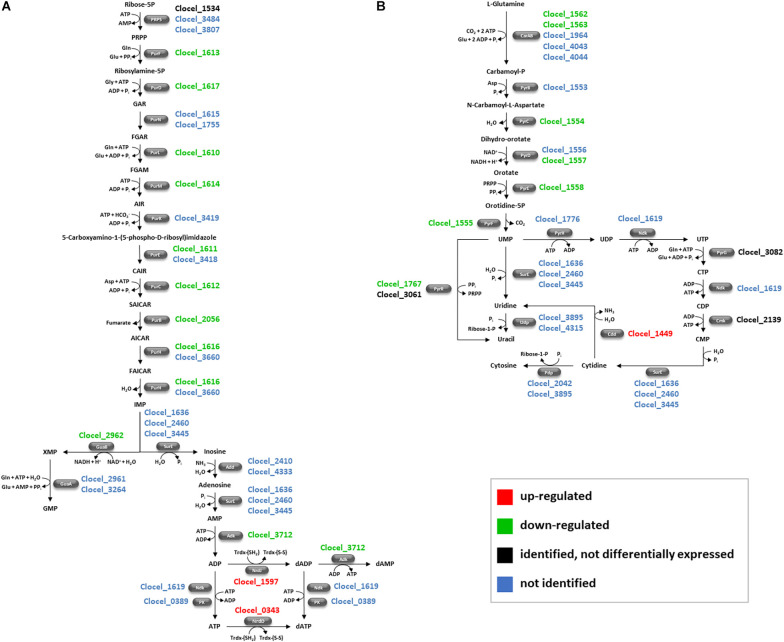
Schematic representation of purine **(A)** and pyrimidine **(B)** metabolic pathways of *C. cellulovorans*. *C. cellulovorans* genes encoding each pathway enzyme is indicated. Red and green colors were used for gene loci whose protein product was over- or under-expressed in butanol-challenged cells, respectively. Geni loci whose protein product was identified in this study but was in similar amounts in butanol-supplemented and control cultures were indicated in black, while blue indicates geni loci whose protein product was not identified in the present investigation. Add, adenosine deaminase; Adk, adenylate kinase; AICAR, 1-(5′-Phosphoribosyl)-5-amino-4-imidazolecarboxamide; AIR, Aminoimidazole ribotide; CAIR, 1-(5-Phospho-D-ribosyl)-5-amino-4-imidazolecarboxylate; CarA, carbamoyl-phosphate synthase, small subunit; CarB, carbamoyl-phosphate synthase, large subunit; Cdd, cytidine deaminase; Cmk, cytidylate kinase; FAICAR, 1-(5′-Phosphoribosyl)-5-formamido-4-imidazolecarboxamide; FGAM, 2-(Formamido)-*N1*-(5′-phosphoribosyl)acetamidine; FGAR, 5*′*-Phosphoribosyl-*N*-formylglycinamide; GAR, 5′-Phosphoribosylglycinamide; GuaA, GMP synthase; GuaB, inosine-5′-monophosphate dehydrogenase; IMP, inosine monophosphate; Ndk, nucleoside-diphosphate kinase; NdrD, anaerobic ribonucleoside-triphosphate reductase; NrdJ, ribonucleoside-diphosphate reductase; P_i_, inorganic phosphate; Pdp, pyrimidine-nucleoside phosphorylase; PK, pyruvate kinase; PRPP, 5-phosphoribosyl 1-pyrophosphate; PRPS, ribose-phosphate pyrophosphokinase; PurB, adenylosuccinate lyase; PurC, phosphoribosylaminoimidazole-succinocarboxamide synthase; PurD, phosphoribosylamine/glycine ligase; PurE, phosphoribosylaminoimidazole carboxylase; PurF, amidophosphoribosyltransferase; PurH, phosphoribosylaminoimidazolecarboxamide formyltransferase/IMP cyclohydrolase; PurK, 5-(carboxyamino)imidazole ribonucleotide synthase; PurL, phosphoribosylformylglycinamidine synthase; PurM, phosphoribosylformylglycinamidine cyclo-ligase; PurN, phosphoribosylamine–glycine ligase; PyrB, aspartate carbamoyltransferase; PyrC, dihydroorotase; PyrD, dihydroorotate dehydrogenase; PyrE, orotate phosphoribosyltransferase; PyrF, orotidine 5′-phosphate decarboxylase; PyrG, CTP synthase; PyrH, uridylate kinase; PyrR, uracil phosphoribosyltransferase; SAICAR, 1-(5′-Phosphoribosyl)-5-amino-4- (*N*-succinocarboxamide)-imidazole; SurE, 5′-nucleotidase; Trdx, thioredoxin; Udp, uridine phosphorylase; XMP, xanthosine 5′-phosphate.

The amount of several enzymes involved in purine (10 proteins) and pyrimidine (7 proteins) biosynthesis was significantly lowered by butanol challenge (Figure 7). This concerns nearly all the pathway enzymes from glutamine to UMP (pyrimidine metabolism, Figure 7B). qRT-PCR analysis seems to further support this evidence since it confirmed down-regulation of the small subunit of carbamoyl-phosphate synthase (Clocel_1562) and dihydroorotase (Clocel_1554) in butanol-stressed *C. cellulovorans* (Table 2). As regards purine metabolism, almost the entire pathway enzymes from phosphoribosyl pyrophosphate (and glutamine) to IMP and then to XMP and AMP were down-regulated (Figure 7A). This picture is similar to that captured by transcriptomic analysis of *C. acetobutylicum* after butanol challenge (Alsaker et al., 2010), although a more recent proteomic analysis of this strain in the same conditions reported an enrichment of proteins involved in purine biosynthesis among overexpressed proteins (Venkataramanan et al., 2015). Down-regulation of enzymes involved in pyrimidine biosynthesis has been reported also in butanol-challenged *Lactiplantibacillus plantarum* while effect on purine pathway enzymes is less pronounced (Petrov et al., 2021). Differential regulation of genes involved in nucleotide biosynthesis has been observed after a number of chemical stresses (e.g., acetate, butyrate, NaCl) in other clostridia (Alsaker et al., 2010; Philips et al., 2017). However, most frequently genes for pyrimidine biosynthesis were down-regulated, while those involved in purine biosynthesis were up-regulated. The reason for differential expression of genes involved in nucleotide metabolism in stress responses still remains elusive. As regards repression of XMP biosynthesis, this could be part of the stringent response mediated by (p)ppGpp, as previously described in *B. subtilis* (Kriel et al., 2012). XMP is an intermediate of the biosynthesis of GTP (Figure 7A), and repression of the pathway for GTP biosynthesis by (p)ppGpp has been observed in *B. subtilis*. This is thought to differentially regulate transcription of genes depending on the initiating nucleotide, possibly by simple mass action (thus GTP depletion would inhibit transcription of genes that start with GTP only). It is worth noting that only enzymes involved in *de novo* biosynthesis of purine and pyrimidine were down-regulated in butanol-challenged *C. cellulovorans* cultures, while, except for the product of Clocel_3712, enzymes catalyzing nucleotide salvage pathways (i.e., that recycle nucleotide precursors obtained by degradation of polynucleotides) were not identified (Figure 7). *De novo* nucleotide synthesis requires higher ATP/GTP expenditure than salvage pathways (Moffatt and Ashihara, 2002). Hence, down-regulation of *de novo* nucleotide synthesis could also be a strategy to reduce energy consumption.

#### Carbohydrate Transport and Metabolism

Butanol challenge induces up-regulation of HPr kinase/phosphorylase (HPrK/P, Clocel_1671), a sensor enzyme involved in the regulation of sugar uptake and carbon metabolism in several bacteria (Deutscher et al., 2014). HPrK/P catalyzes the ATP- and PP_i_-dependent phosphorylation of Ser46 of HPr, a protein of the PEP-dependent sugar phosphotransferase system (PTS), and also its dephosphorylation (Fieulaine et al., 2001). Conditions leading to increase of fructose 1,6-bisphosphate (F1,6BP) concentration activate kinase activity of HPrK/P which improves the level of phosphorylation of HPr-Ser46 (Deutscher et al., 2014). P-Ser46-HPr acts as a co-regulator (mainly a co-repressor) of gene transcription (e.g., genes involved in carbohydrate transport and catabolism, including glycolysis) (Shimizu and Matsuoka, 2019). On the other hand, increase in intracellular levels of P_i_ inhibits kinase activity of HPrK/P and stimulates its phosphorylase activity (Fieulaine et al., 2001). In addition, a putative glucose PTS transporter (Clocel_2778) is up-regulated in butanol-challenged *C. cellulovorans*. The latter result seems consistent with increased specific glucose consumption observed in cells grown in butanol-supplemented conditions. However, three glycolytic enzymes, namely phosphoglycerate kinase (Clocel_0720), triose phosphate isomerase (Clocel_0721) and pyruvate phosphate dikinase (Clocel_1454) were down-regulated in butanol-challenged *C. cellulovorans*. Interestingly, up-regulation of some/most key glycolytic enzymes was reported in other bacteria (e.g., *C. acetobutylicum*, *S. warneri*) in response to butanol stress (Fu et al., 2013; Venkataramanan et al., 2015).

#### Central Carbon Metabolism and Fermentative Pathways

The amount of some key enzymes involved in *C. cellulovorans* fermentative pathways (e.g., acetate, butyrate and formate production) was significantly altered by butanol exposure.

Acetate kinase (Ack, Clocel_1892, acetyl phosphate + ADP → acetate + ATP) was two-fold overexpressed in butanol-supplemented cultures, which is consistent with increased acetate yield observed in this condition (Figure 2). It is tempting to speculate that improved acetate production could be related to increased ATP requirement under alcohol-stressed conditions. A slight Ack up-regulation was observed also in butanol-stressed *C. acetobutylicum* (Alsaker et al., 2010).

As mentioned above, a statistically significant decrease in formate yield was observed in butanol-challenged *C. cellulovorans* (Figure 2). Proteomic analysis actually showed 2-fold down-regulation of pyruvate formate lyase (PFL)-activating enzyme (Clocel_1812) in butanol-challenged cells. Down-regulation of PFL after butanol challenge was observed also in *C. acetobutylicum* (Tomas et al., 2004; Alsaker et al., 2010). In addition, a pyruvate ferredoxin oxidoreductase (PFOR, Clocel_1684) and a hydrogenase large subunit (Clocel_3813) were twofold overexpressed in butanol-grown *C. cellulovorans*. Previous studies have suggested that Clocel_1684 likely encodes the main PFOR of *C. cellulovorans* (Ou et al., 2019; Usai et al., 2020). Its up-regulation also supports the hypothesis that a higher amount of pyruvate is metabolized through PFOR instead of PFL reaction in butanol-exposed cells. Since in the PFOR reaction, pyruvate is oxidized with concomitant reduction of ferredoxin, a higher amount of reduced ferredoxin would be available for hydrogen production in these growth conditions. *C. cellulovorans* genome encodes four hydrogenases. Interestingly, butanol-exposure induced up-regulation of only the product of Clocel_3813 while that encoded by Clocel_4097 was found to be up-regulated in glucose-grown *C. cellulovorans* (Ou et al., 2019; Usai et al., 2020). Anaerobic bacteria generally have multiple hydrogenases whose specific function is often unclear (Mazzoli, 2012). It is therefore difficult to determine the precise relationship between these particular hydrogenases and changes in their expression depending on environmental conditions. Interestingly, a similar modulation of the pyruvate node after butanol shock was reported in *C. acetobutylicum* featuring down-regulation of PFL and overexpression of a PFOR and a hydrogenase (Alsaker et al., 2010; Venkataramanan et al., 2015).

**FIGURE 8:**
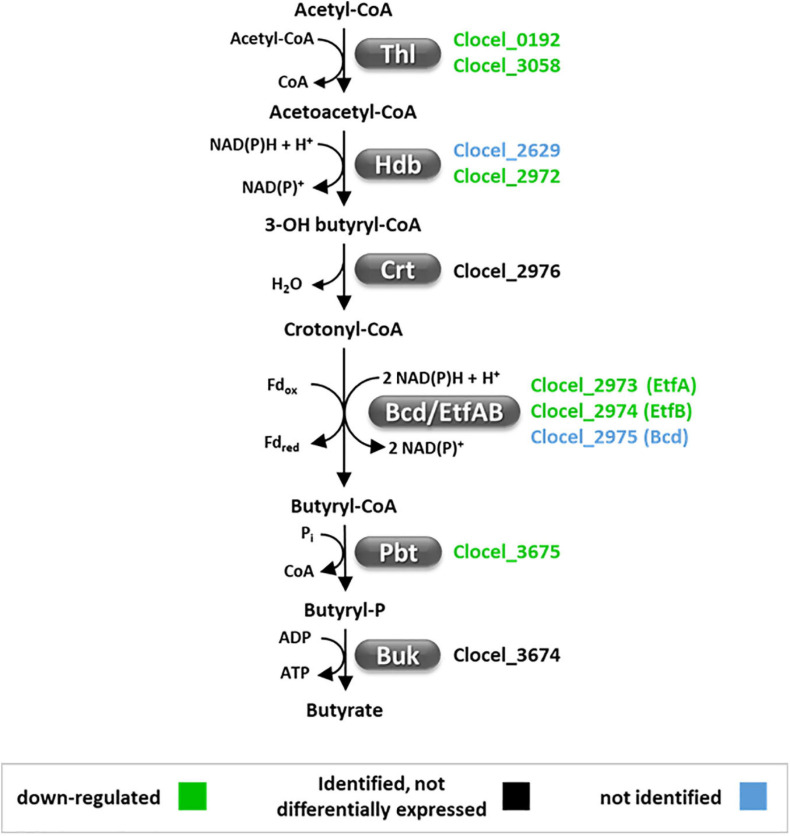
The acetyl-CoA–butyrate pathway is down-regulated in butanol-challenged *C. cellulovorans*. Gene loci encoding the pathway enzymes are represented in colors indicating their level of relative expression in butanol-supplemented cultures (green, down-regulated; black, identified but not differentially expressed, blue, not identified). Abbreviations: Bcd/EftAB, butyryl-CoA dehydrogenase/electron transfer protein; Buk, butyrate kinase; Crt, crotonase; Fd, ferredoxin; H_2_ase, hydrogenase; Hbd, 3-hydroxybutyryl-CoA dehydrogenase; Pbt, phosphate butyryltransferase; Thl, thiolase.

Proteomic analyses indicated that butyrate production pathway is inhibited in butanol-challenged *C. cellulovorans*. Six gene products (i.e., acetyl-CoA acetyltransferases, Clocel_0192 and Clocel_3058; 3-hydroxybutyryl-CoA dehydrogenase, Clocel_2972; electron transfer flavoprotein α and β subunits, Clocel_2973 and Clocel_2974; and phosphate butyryltransferase, Clocel_3675) out of ten involved in acetyl-CoA conversion to butyrate were down-regulated (Figure 8). In addition, slight down-regulation of enoyl-CoA hydratase/isomerase (Clocel_2976) and butyrate kinase (Clocel_3674), although not statistically significant, provided a further evidence that the butyrate production pathway is repressed by butanol supplementation in *C. cellulovorans*. These data are inconsistent with increase in butyrate yield observed in butanol-challenged *C. cellulovorans* (Figure 2). However, carbon flux through a pathway is not solely controlled by the concentration of pathway enzymes. Down-regulation of the butyrate pathway may have contributed to divert a higher proportion of acetyl-CoA toward acetate production and possibly fatty acid biosynthesis. Acetate/butyrate ratio was actually increased by 50% in butanol-challenged cultures (Figure 2). Genes Clocel_2972-2976 form a cluster for butyryl-CoA synthesis which is similar to that found in other clostridia such as *C. kluyveri* and *C. acetobutylicum* and was demonstrated to be under the control of the redox-responsive transcriptional regulator Rex (Hu et al., 2016). Actually, upstream of Clocel_2972-2976 is located a gene (Clocel_2977) which encodes a protein that shares 71% sequence identity with *C. acetobutylicum* Rex. A search for the *Clostridium* Rex DNA binding element (TTGTTAANNNNTTAACAA) identified two motif instances upstream (–23 and –88 from Clocel_2976 transcription start site) of the Clocel_2972-2976 gene cluster further supporting the hypothesis that Rex regulates its expression. Additional putative Rex binding motif instances were found upstream of acetyl-CoA acetyltransferase encoding genes (Clocel_0192 and Clocel_3058) but not upstream of genes encoding butyrate kinase and phosphate butyryltransferase (Clocel_3674 and Clocel_3675, respectively). These data suggest that Rex may be involved in the regulation of gene expression in butanol-challenged *C. cellulovorans*. Interestingly, it was recently reported that the Rex regulon was not affected by butanol stress in *C. acetobutylicum* (Venkataramanan et al., 2015). However, up-regulation of the butyryl-CoA formation operon (*bcd*, *etfAB*, *crt*) and thiolase after butanol shock was reported, which corresponds to an opposite trend with respect to what observed in the present study on *C. cellulovorans*. It is worth remembering that Rex is a gene transcription repressor whose activity depends on low intracellular NADH/NAD^+^ ratio (Ravcheev et al., 2012). These results therefore indicate a different regulation of the butyryl-CoA formation operon between *C. cellulovorans* and *C. acetobutylicum* which may reflect different redox states, namely a more oxidized cell status for *C. cellulovorans*.

In addition, two alcohol dehydrogenases (ADHs, encoded by Clocel_4197 and Clocel_1949) were two-fold overexpressed in butanol-enriched cultures. The *C. cellulovorans* genome encodes seven putative ADHs whose function has not been determined in detail yet. Interestingly, the products of Clocel_4197 and Clocel_1949 were not identified in the proteome of glucose- or avicel-grown *C. cellulovorans* which suggests that they are not biosynthesized in high amounts in physiological conditions (Usai et al., 2020). A number of studies have identified ADHs as involved in conferring alcohol resistance to bacteria since they were overexpressed under alcohol-stress (Rutherford et al., 2010) or their deletion or mutation in cofactor (NADH/NADPH) specificity improved alcohol tolerance (Brown et al., 2011; Dai et al., 2012; Tian et al., 2019; Mazzoli and Olson, 2020). Recently, butanol dehydrogenase activity has been hypothesized for the product of both Clocel_4197 and Clocel_1949 (Wen et al., 2019) although this requires experimental confirmation. However, no significant butanol consumption by *C. cellulovorans* was detected in the present investigation (data not shown). Currently, an explanation for the up-regulation of these proteins in butanol-challenged *C. cellulovorans* is therefore elusive. A more detailed characterization of *C. cellulovorans* ADHs, with particular attention on those encoded by Clocel_4197 and Clocel_1949, aimed at determining their substrate(s), cofactor(s) and metabolic role(s) seems important for improving understanding of *C. cellulovorans* physiology and strategies to tolerate butanol.

#### Oxidative Stress Response and Redox Balance

Overexpression of proteins involved in oxidative stress response under solvent (e.g., ethanol, butanol) stress has been observed in different microorganisms (Rutherford et al., 2010; Zhu et al., 2013; Venkataramanan et al., 2015; Charoenbhakdi et al., 2016). Proteins up-regulated by butanol in *C. cellulovorans* include a desulfoferrodoxin (Clocel_4154) and a nitroreductase (Clocel_4148). Desulfoferredoxin catalyzes NAD(P)H-dependent reduction of superoxide anion to hydrogen peroxide (Riebe et al., 2007) and was found among up-regulated proteins also in butanol-challenged *C. acetobutylicum* (Alsaker et al., 2010). Nitroreductase, a flavoprotein that catalyzes NAD(P)H-dependent reduction of substrates, has sometimes been associated with oxidative stress response (Roldán et al., 2008; Rutherford et al., 2010; Williams et al., 2015).

In addition, two enzymes of the tricarboxylic (TCA) cycle, namely citrate synthase (Clocel_3688) and isocitrate dehydrogenase (Clocel_2469) were up-regulated in butanol-challenged *C. cellulovorans*. Up-regulation of isocitrate dehydrogenase was reported as part of the butanol response of other gram positive bacteria (Alsaker et al., 2010; Fu et al., 2013). The function of the TCA cycle in *Clostridia* has mainly been associated with production of intermediates for biosynthetic routes and regulation of the redox balance (namely, isocitrate dehydrogenase reaction generates NADH) (Shinohara et al., 2013).

### Determination of Cellular ATP Content

**FIGURE 9:**
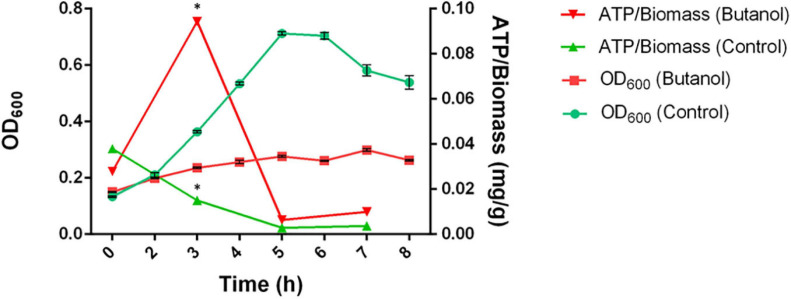
Growth curve and intracellular ATP content of *C. cellulovorans* grown in control condition (green) or in butanol-supplemented medium (red). Bars represent standard deviations (*n* = 3). Asterisks indicate values that significantly (^∗^*p*-value < 0.01) differ between the two growth conditions.

Solvents, including butanol, are generally thought to decrease cellular ATP concentration by a number of mechanisms, namely by: (i) increasing membrane permeability to ATP; (ii) inhibiting membrane-bound ATPases; (iii) increasing membrane permeability to protons and other ions thus leading to dissipation of the proton motive force; (iv) inducing energy-consuming adaptation mechanisms (e.g., efflux systems) (Bowles and Ellefson, 1985; Heipieper et al., 2007). In addition, the present study revealed that a number of proteins directly involved in ATP synthesis (acetate kinase and several subunits of both V-type and F-type ATPases) were up-regulated in butanol-challenged *C. cellulovorans*. However, determination of cellular ATP content showed that ATP levels were generally similar in control- and butanol-supplemented cultures of *C. cellulovorans* (Figure 9). Interestingly, a transient significant increase of ATP levels was observed in butanol-challenged *C. cellulovorans* 3 h after inoculum. Although an explanation for this observation currently remains elusive, a similar phenomenon was previously reported in *E. coli* in response to temperature upshift (Soini et al., 2005). It is worth remembering that transient increase of ATP concentration in *E. coli* did not correspond to improvement of energy charge but rather to a decrease of this parameter, since a higher increase of ADP level occurred concomitantly. It has been speculated that transient ATP level increase in *E. coli* could be a strategy to cope with higher ATP consumption for protein- and DNA-repair mechanisms (Soini et al., 2005). Although solvent effects on the activity or expression of proteins involved in ATP synthesis/consumption have been reported by several studies (Heipieper et al., 2007; Cao et al., 2017), direct determination of ATP content of cells exposed to solvents have seldom been determined. In ethanol-challenged *Arthrobacter simplex*, a decrease of ATP content with respect to control conditions was observed for ethanol concentrations higher than 4% (v/v) (Luo et al., 2018). However, in the solvent-tolerant bacterium *Pseudomonas putida* DOT-T1E, no significant difference in ATP content was detected during fermentation with or without 1-decanol (Neumann et al., 2006). It has been hypothesized that maintenance of unchanged levels of ATP and metabolic energy in *P. putida* in the presence of solvents was likely obtained at the expense of reduced growth efficiency. This explanation could also apply to the present observations on *C. cellulovorans*.

## Conclusion

The results obtained by the present investigation indicate that butanol exposure elicits complex responses in *C. cellulovorans* thus confirming previous observations made in other microorganisms. These responses encompass adaptation mechanisms at the cell wall, cell membrane and cytoplasmic levels:

• Butanol-challenged *C. cellulovorans* dedicated a massive effort in the biosynthesis of proteins involved in protein translation, folding and degradation (28% of the up-regulated proteins). However, lower total cell protein levels measured in butanol-supplemented *C. cellulovorans* cultures (Figure 5) seems more consistent with the fact that protein translation is inhibited in this condition (possibly by ribosome inactivation through the ribosome hibernation promoting factor, Clocel_3036). This is coherent with observations made on other butanol-stressed clostridia (Alsaker et al., 2010; Venkataramanan et al., 2015; Sedlar et al., 2019) as well as other butanol-challenged bacteria (Fu et al., 2013; Tian et al., 2013). It remains to be determined why a large number of ribosomal subunits were up-regulated in butanol-supplemented *C. cellulovorans* cultures.
• Proteomic data suggest that a re-arrangement of both the cell wall (i.e., peptidoglycan) and membrane composition occurs in butanol-stressed *C. cellulovorans*. As regards cell membrane, our findings suggest that a higher proportion of saturated fatty acids is incorporated in the cell membrane in this condition thus decreasing membrane fluidity, consistently with observations made in other microorganisms (Borden and Papoutsakis, 2007; Huffer et al., 2011; Isar and Rangaswamy, 2012; Fu et al., 2013).
• Many membrane transport proteins, and in particular ion transporters possibly involved in ATP synthesis and/or generation of transmembrane electrochemical gradient were up-regulated in butanol-challenged *C. cellulovorans*. These proteins include Na^+^ (NatB, Clocel_3460) and Mg^2+^ (MgtE, Clocel_1328) transporters and several subunits of a V-type ATPase (V_1_ subunits K, Clocel_1656; C, Clocel_1658; F, Clocel_1659; A, Clocel_1660; and B, Clocel_1661) and a F-type ATPase (F_1_ subunits γ, Clocel_3050; α, Clocel_3051; and δ, Clocel_3052).
• Multiple metabolic pathways were significantly affected by butanol exposure including: (i) up-regulation of enzymes involved in amino acid biosynthesis; (ii) down-regulation of several enzymes involved in pyrimidine and purine biosynthesis; (iii) modulation of the expression of multiple enzymes involved in glycolysis and fermentative pathways. As regards amino acid biosynthesis, the enhancement of enzymes catalyzing biosynthesis of branched-chain amino acids (acetolactate synthase large subunit, Clocel_1324, ketol-acid reductoisomerase, Clocel_1325, and dihydroxy-acid dehydratase, Clocel_0493) could possibly refer to additional mechanisms to modulate cell membrane fluidity (Mansilla et al., 2004). In fact, branched-chain amino acids are used as primers for the synthesis of branched-chain fatty acids (Mansilla et al., 2004; Alsaker et al., 2010; Wang et al., 2016). Inconsistent with studies reporting butanol inhibition of glucose uptake in other bacteria (Bowles and Ellefson, 1985; Tomas et al., 2004; Alsaker et al., 2010; Venkataramanan et al., 2015; Sedlar et al., 2019), butanol caused an increase of the specific glucose consumption in *C. cellulovorans*. This observation possibly correlates with the up-regulation of a putative glucose PTS transporter (Clocel_2778). In addition, pyruvate fate was significantly perturbed by butanol-stress in *C. cellulovorans*. Formate production was inhibited (consistent with down-regulation of PFL-activating enzyme, Clocel_1812), and a higher proportion of the acetyl-CoA was driven to acetate instead of butyrate (Figure 2). The latter observation correlates well with the down-regulation of the acetyl-CoA to butyrate pathway (i.e., acetyl-CoA acetyltransferase, Clocel_0192 and Clocel_3058; 3-hydroxybutyryl-CoA dehydrogenase, Clocel_2972; electron transfer flavoprotein α and β subunits, Clocel_2973 and Clocel_2974; and phosphate butyryltransferase, Clocel_3675) (Figure 8). Although the metabolic reason for this re-arrangement is currently unclear, one can hypothesize that down-regulation of the butyrate pathway may also serve to drive more acetyl-CoA toward fatty acid biosynthesis.

The present study has highlighted several potential gene targets for improving butanol tolerance of *C. cellulovorans* through rational metabolic engineering. The complexity of microbial responses to butanol stress makes identification of priority gene modifications aimed at these strategies not trivial. Furthermore, a significant number of previous studies reported involvement of genes with previously unknown/poorly characterized function in butanol tolerance (Jia et al., 2012; Pei et al., 2017; Sun et al., 2017). Although an exhaustive mechanistic understanding of microbial adaptation to butanol stress currently remains elusive, several studies have obtained significant improvement of butanol tolerance by single/few gene modifications (Anfelt et al., 2013; Si et al., 2016; Yang et al., 2020) which may serve as general paradigms for these strategies. So far, most examples of targeted gene modification aimed at enhancing butanol tolerance have been based on overexpression of HSPs (e.g., GroES, GroEL, and DnaK) which proved to be effective in very diverse microbial models such *E. coli* (Zingaro and Papoutsakis, 2012), lactobacilli (Fiocco et al., 2007), cyanobacteria (Anfelt et al., 2013), and clostridia (Tomas et al., 2003). On the basis of the high fold changes measured by the present proteomic analysis, the most interesting *C. cellulovorans* gene targets include *groES* (Cpn10, Clocel_2966, FC = 5.05), *htpG* (Clocel_0510, FC = 3.99), *dnaJ* (Clocel_1417, FC = 3.32) and *clpC* (ATPase AAA-2 domain protein, Clocel_3760, FC = 2.80). Recently, an increasing attention has been devoted to the role of sRNAs in response to a variety of stresses including butanol, and studies involving overexpression or suppression of some of them were effective in improving butanol tolerance (Venkataramanan et al., 2013; Jones et al., 2016; Sun et al., 2017). These data seem to find confirmation in *C. cellulovorans* since the RNA chaperone Hfq (Clocel_2035) was among the most up-regulated protein under butanol-stress (FC = 3.09). Therefore, overexpression of Hfq is another interesting strategy that would be worth testing. Recently, overexpression of several sRNA regulators was used to improve butanol-tolerance of *E. coli* (Xu et al., 2020). A further obvious strategy concerns overexpression of proteins involved in maintaining homeostasis of the transmembrane Δψ and of ATP concentration such as the V-type (Clocel_1654-1662) and F-type (Clocel_3048-3055) ATPases identified in this study.

It is worth noting that the function of four among the ten most up-regulated proteins in butanol-stressed *C. cellulovorans* (FC ≥ 3.5) is currently unknown. Our study thus points at interesting gene candidates for engineering butanol-hypertolerant *C. cellulovorans* and stimulates further studies aimed at characterizing still poorly characterized proteins.

## Data Availability Statement

The datasets presented in this study can be found in online repositories. The names of the repository/repositories and accession number(s) can be found in the article/Supplementary Material.

## Author Contributions

RM conceived the study. PC and GU performed the bacterial cultures and prepared extracts for proteomic analysis. MM performed the proteomic analysis. PC, GU, and AR analyzed the proteomics results. RM, GM, and CB performed the qRT-PCR analysis. PC, GU, AR, MM, GM, CB, and RM prepared the figures and tables. PC, GU, AR, CB, EP, and RM interpreted the results. All the authors contributed to writing the manuscript.

## Conflict of Interest

The authors declare that the research was conducted in the absence of any commercial or financial relationships that could be construed as a potential conflict of interest.

## Footnotes

1 http://eggnogdb.embl.de/#/app/home

2 https://blast.ncbi.nlm.nih.gov/Blast.cgi?PAGE=Proteins

3 https://www.uniprot.org/uniprot/
