# Supplementary material for: Clostridium cellulovorans Proteomic Responses to Butanol Stress
Source: Front Microbiol. 2021 Jul 21;12:674639. doi: 10.3389/fmicb.2021.674639 (PMC8336468; doi:10.3389/fmicb.2021.674639)
Supplement: Supplementary file 1 [file Table_1.DOCX]

**Supplementary Table 1.** Primer sequences used in quantitative real-time PCR analysis

| **ID** | **Locus tag** | **Description** |  | **Sequence** |
| --- | --- | --- | --- | --- |
|  |  | **Reference gene** |  |  |
| ADL53404.1 | Clocel_3734 | *RpsJ/30S ribosomal protein S10* | **F** | TCAACAGTTTTTGGTGATGGA |
|  |  |  | **R** | CCAGTGCCACTACCAACTGA |
|  |  | **Target genes** |  |  |
| ADL51618.1 | Clocel_1874 | *Methionyl-tRNA formyltransferase* | **F** | AATTCGCGGTTCCTTCATTA |
|  |  |  | **R** | TTCCATGTTCAAGGGCTACC |
| ADL52726.1 | Clocel_3036 | *Sigma 54 modulation protein/ribosomal protein S30EA* | **F** | ACTGCTTCCTCAGCGTTCAT |
|  |  |  | **R** | TGCATTATCAAAGGGGGAAC |
| ADL51814.1 | Clocel_2071 | *(p)ppGpp synthetase I, SpoT/RelA* | **F** | CCATTAACCTTAGCCCCGATA |
|  |  |  | **R** | CCTGCGGGAAAAGTAATCAA |
| ADL52658.1 | Clocel_2966 | *Chaperonin Cpn10* | **F** | TTTGTCTCCCACTTTTAATTCCA |
|  |  |  | **R** | CAGCGAAGGAAAAACCTCAG |
| ADL50285.1 | Clocel_0510 | *Heat shock protein Hsp90-like* | **F** | CCTTGACGAGAACCTTGGAG |
|  |  |  | **R** | TCCGCTACCATAAATGCAGA |
| ADL53559.1 | Clocel_3893 | *Asparagine synthase (glutamine-hydrolyzing)* | **F** | CCAAACATTTCATCGGAACC |
|  |  |  | **R** | ACATGGACACATTGCCAAAA |
| ADL53431.1 | Clocel_3761 | *ATP:guanido phosphotransferase* | **F** | ATTTCCATGCGCTTTTGAAC |
|  |  |  | **R** | CAGGAATGAGAGCTTCAGCA |
| ADL54009.1 | Clocel_4352 | *Acyl-ACP thioesterase* | **F** | TTTTGGTTTTGGCATTTTGA |
|  |  |  | **R** | GCAATGGAGATGAGATAGCAAA |
| ADL51400.1 | Clocel_1656 | *H^+^transporting two-sector ATPase C subunit* | **F** | GGAAAAGCAGCAGCTAGTGG |
|  |  |  | **R** | TGCAACGAAACCGAGTAGTG |
| ADL51404.1 | Clocel_1660 | *H^+^transporting two-sector ATPase alpha/beta subunit central region* | **F** | AGTGGCCGGTAAGAAAAGGT |
|  |  |  | **R** | TTTTGCAACTGGGAAAAAGG |
| ADL51310.1 | Clocel_1562 | *Carbamoyl-phosphate synthase, small subunit* | **F** | GCAAAGCTTTTGGAAAGCAA |
|  |  |  | **R** | ATCTGCCCTGCATAAGATGG |
| ADL51302.1 | Clocel_1554 | *Dihydroorotase, multifunctional complex type* | **F** | ATCGCTCTTACGGGAGAAGG |
|  |  |  | **R** | CCTTTGTATGTGGTGCATGG |
| ADL51081.1 | Clocel_1328 | *MgtE intracellular region* | **F** | ATCCAGCAGACCTTGCAGAT |
|  |  |  | **R** | CCTGGCTCGATTTCCTCTAA |
